# ‘It is like talking to very good robots’: Experiences of online support groups for parents with babies during the COVID-19 lockdown in the United Kingdom

**DOI:** 10.1177/14733250231185066

**Published:** 2023-06-21

**Authors:** Siân E Lucas, Laura Bellussi

**Affiliations:** Faculty of Social Sciences, Centre for Child Wellbeing and Protection, 7622 University of Stirling, Stirling, UK

**Keywords:** New parents, newborns, postnatal support, perinatal support, parent groups, online groups, digital parenting

## Abstract

During the COVID-19 pandemic, many face-to-face group activities for new parents moved online. In this article, we share findings from 14 interviews conducted during the first lockdown in the United Kingdom with parents of babies under 12 months about their experiences of participating in online groups. Attendance at groups was treated as a survival mechanism or even a panacea for some parents, providing well-rounded entertainment and support for parents, their children and the parenting relationship. However, reviews of the online groups were mixed, with some deemed more adequate than others for online delivery. Parents expressed concerns about the lack of informal spaces in online contexts to share private conversations and the unnatural group conversations affected by the custom of taking turns to talk. These experiences highlighted what was important to parents: the need for the embodiment of connection. The peculiarity of the online setting saw the emergence of parenting display work; parental awareness of online impression management and self-presentation strategies. While online technologies are ubiquitous, online groups for parents are nascent, and these technologies require careful analysis and evaluation from users and facilitators.

## Introduction

### Early childhood and the postnatal period

The first 1000 days of a child’s life constitute a critical window, which yields the potential to provide a solid foundation for lifelong growth and development (Moore et al., 2017). During the early years, the foundations of sensory, motor and perceptual systems critical to language, social behaviour, emotions, and cognitive and physical development are formed and are strongly influenced by experience (Clark, 2005; Tierney & Nelson III, 2009). The early childhood years are also crucial for developing healthy and positive attachment bonds with primary caregivers.

### Parenting support in the postnatal period

In this article, we consider the postnatal period as 12 months after childbirth, a timeframe widely accepted as a crucial period of transition for babies’ development and the development of new parent identities (Heinicke et al., 1983; Rochat and Striano, 1999; Fahey and Shenassa, 2013). Perinatal is an all-encompassing term which refers to the experience of pregnancy, labour and the period after birth, whereas postnatal refers to the process after birth. In this article, while we refer to parents and mainly mothers, we recognise that there are lots of ways to compose a family beyond biological and maternal relationships.

The basic form of support provided to new parents in the postnatal period is ‘parental leave’, also known as ‘family leave’: a period off work granted to a caregiver to care for a child. There are different forms of parental leave, including maternity leave, paternity leave and adoption leave, and the length and legal conditions vary across countries (Addati et al., 2014). During parental leave, time-space behaviours and everyday activities are reorganised and restructured to allow parents to meet their own and their child’s caregiving needs and responsibilities (Dyck, 1990).

During parental leave, many mothers and fathers join parents and babies’ groups, which can be defined as ‘a site of knowledge production and repository of experience’ (Hickey, 2019, p. 483). Research has shown that parent groups are essential during the postnatal period. Groups provide a way to access advice and share everyday experiences and problems with peers (Frantál and Klapka, 2019). They are also beneficial for decreasing social isolation, combatting loneliness, fatigue and adjusting to life with a new baby (Hickey, 2019; Killgore et al., 2020). Postnatal groups have been found to support parents in multiple ways, including support to continue breastfeeding and minimise postnatal depression (Scottish Government, 2017; Barlow et al., 2012).

Before the COVID-19 restrictions came into effect in the United Kingdom in 2020, groups for new parents were typically offered in an in-person format and included activities to benefit parents and babies and their bonding relationship; such as singing and rhyming, storytelling, signing, baby massage, baby sensory, yoga, swimming and buggy walks amongst others. The advent of physical distancing measures brought newfound reliance on digital technologies and resulted in emergent online parent groups.

### Digital parenting

Before the pandemic, digital technology was already a prominent feature in contemporary life and rather than copying or replacing the ‘real world’, online interactions, virtual social support and networks were found to ‘supplement’ parents’ lives and offer temporary relief from physical meetups (Madge and O’Connor, 2006). In this article, we explore the way that online groups functioned beyond a ‘supplement’ to become a ‘lifeline’ for parents.

New mothers are known to be among the most active users of Facebook (Maslen, 2022). Social media platforms have attracted attention for their mobilising abilities, yet also have affective and emotional implications, as their interactional practices include empathy, love and affection (Brownlie and Shaw, 2019; Fussey and Roth, 2020). Participating about parenting practices in online spaces, can provide users with social and emotional support and validation of ‘good mothering practices’ (Chan, 2008). The messaging app ‘WhatsApp’ is often used by groups of mothers who create a pool of parenthood-related expert and experiential knowledge to support one another through their babies’ early years (Lyons, 2020).

### Digital presentation

Finch (2007) uses the notion of ‘displaying families’, and argues that *‘families need to be ‘displayed’ as well as ‘done*’ (original italics: 66); this is because the meaning of actions are both conveyed to and understood by others if those actions are to be effective in constituting family practices. The presentation of such meanings becomes subject to special interest when families and parenthood are ‘displayed’ through an online lens. Madge and O’Connor (2005) argue that anonymous online interaction can help mothers adjust to new ways of life and their changing identity. Indeed, Bargh et al. (2002) found that internet interactions allowed individuals to express their ‘true selves better’, including aspects of themselves that they wanted to express but felt unable to in face-to-face interactions.

On the other hand, Robinson (2007) argues that individuals are subjected to specific social pressures and constraints in presenting an online self. Extending this idea, Broadbent and Lobet-Maris’s study (2015) found that online users were in ‘pursuit of a protective cocoon’, resulting in an extreme form of filtering social and relational information. Goffman’s dramaturgical theory (1959) has been used by various researchers to investigate how people form their social identities on social media networks. Goffman argues that ‘performances’ take place in the front and back regions; in the front region, the individual plays a certain role – defined by appearance, the stage and the manner of performance. In the back region, individuals may perform more informally and sometimes in a way which may contradict their performance in the front ‘stage’. It is argued that in social media presentations, the user has control of their self-presentation in terms of the content they publish and the role they undertake. Merunková and Šlerka (2019) present research to suggest that Facebook is used as a ‘self-presentation platform’ where, in the front region, users create public posts in selective ways to present their desired identity. However, messages and group chats are back region spaces, where people discuss content that could not be shared publicly, which may be negative, serious or contradictory to what is publicly presented in the front region.

In 2013, Chambers (2013) claimed that in a time of rapid and complex technological change, technology was supplanting and absorbing different communication types to the extent to which friendships and family relationships had been reconfigured by technology. Indeed, for families who live some or most of the time separately, digital technologies have been used to maintain transnational connections (Guyer et al., 2000; Longhurst, 2013; Francisco, 2015). Technologies also create opportunities for ‘latent ties’, facilitating contact between people, which would not otherwise be possible (Haythornthwaite, 2005). There is consensus that, like many domains, technologies became essential for parents’ wellbeing, during the pandemic, in times of physical distancing restrictions.

### The impact of lockdown during the perinatal period

Pregnancy and early motherhood are times of unparalleled contact with health services (Royal College of Psychiatrists, 2021); however, across the United Kingdom, perinatal services were deeply affected by the pandemic, and healthcare services were reorganised to minimise the spread of the virus. Telehealth, videoconferencing, recordings, text messaging, smartphone apps and online chats were some of the methods used in healthcare settings across the world to reduce in-person interaction and mitigate the risk of infection.

The attendance of birth partners was restricted at antenatal appointments, scans, labour and in neonatal settings (Scottish Government, 2021; Vazquez-Vazquez et al., 2021). In Poland, severely sick or prematurely born babies were separated from their mothers due to restriction protocol (Wszołek et al., 2022). In Scotland, health visitors (nurses or midwives who have undertaken further study and who work in the community to support families with young children) were redeployed within the National Health Service (NHS), meaning that routine health checks and appointments for mothers and their babies were cancelled.

Chen et al. (2021) argue that despite the perceived benefit of minimising infection by reducing physical interaction, the dearth of human connection was problematic, and patients’ and healthcare providers’ relationships were compromised. Molgora and Accordini (2020) argue that women’s limited access to formal and informal support networks created new struggles and had a negative impact on expectant and postpartum women’s wellbeing; increasing social isolation and decreasing support and women’s autonomy in health care. Parents who gave birth during the lockdown without their birth partners present said it was impossible to compensate for this absence (Baran et al., 2021).

Brown and Shenker (2020) distributed an online survey, which was completed by 1219 breastfeeding mothers to babies aged up to 12 months in the United Kingdom, and found that the pandemic had affected women’s breastfeeding experiences in two key ways: 41.8% of mothers felt that breastfeeding was protected due to lockdown and increased time at home, less pressure and fewer visitors. However, 27% of mothers struggled to get support, worrying about the safety of feeding and feeling isolated; they experienced numerous barriers, and some stopped breastfeeding before they were ready, directly blaming the impact of the pandemic.

The postnatal period is associated with substantially increased risks of severe mental illness and psychiatric admission (Thapa et al., 2020; Royal College of Psychiatrists, 2021). Higher proportions of poorer health have been reported during the lockdown across all outcomes amongst caregivers compared to non-caregivers, with psychological distress commonly reported (Park, 2020). In a Spanish study, women who gave birth during the pandemic perceived a higher level of stress and a poorer quality of care (Mariño-Narvaez et al., 2021). Suárez-Rico et al. (2021) report that the prevalence of depressive symptoms, generalised anxiety and perceived stress was higher among postpartum Mexican women during the COVID-19 outbreak than before the lockdown. Fernandes et al. (2021) indicate that during the lockdown periods, new mothers may have been more focused on fear and danger about the unknown disease and its consequences, and this may have led some Portuguese mothers to decentre from their own emotions, which negatively affected parent: infant bonding.

Das, 2020, 2021 carried out qualitative research with 14 pregnant women and new mothers in the United Kingdom during the 2020 lockdown and reported that participants experienced significant levels of exhaustion or isolation coping with perinatal challenges amidst the pandemic. Online communities and informal online connections offered women respite and camaraderie; however, there were limits to perinatal support offered online, including inadequacies in internet connection, poor call quality and interruptions to conversations. Limited access to formal and informal support networks during the lockdown period meant that pregnant and postpartum women’s mental health was in jeopardy. On the other hand, Fontanesi et al. (2020) recognised that lockdown might include positive factors for parents, such as the opportunity to spend more time with their family and children.

It is clear from the literature that the social isolation and reduction of services due to the COVID-19 pandemic presented challenges for people during the perinatal period. The online delivery of parental groups and classes might have mitigated some of these troubles, but also added further difficulties because of the peculiarity of the online setting, which could impact the way parents presented themselves and interacted with their peers. Building from the existing evidence, we designed a qualitative study to address the following research questions.

1. What are new parents' experiences of using online parental groups and classes during the first COVID-19 lockdown in the United Kingdom?
2. How has parents’ increased reliance on technology affected their ways of accessing support and interacting socially in such contexts?

## Methodology

This study was granted an expedited ethics review by the General University Ethics Panel of the University of Stirling in April 2020, justified by our interest in exploring in real-time the experiences of new parents during the first COVID-19 pandemic. Individuals were invited to participate in remote interviews if they were parents of babies up to 12 months of age living in the United Kingdom between May and June 2020 and had attended online parent groups. The study was advertised on our personal Twitter profiles and on Facebook parent groups located in Scotland between May and June 2020: posters about the study were included, providing details about how to contact the researchers and allowing other social media users to share them.

Participant information sheets were given to prospective participants. 20 people responded to the online advert, and 14 individuals agreed to be interviewed. Reasons for withdrawals are unknown and have not been explored, but given the circumstances of the lockdown, we hypothesised that parental and/or work duties might have affected their availability or interest.

Participants gave written consent and completed an online demographic questionnaire to identify their gender, ethnicity, age, household income and postcode. Interviews lasted up to 1 hour and took place via video call on Microsoft Teams or over the phone, based on participants’ choice. The interviews were semi-structured and based on a pre-determined topic guide, which aimed to explore participants’ experiences of attending online groups. No incentives were given to participants for their involvement.

Interviews were recorded with permission and lasted between 18 and 57 min, with an average of 32 min. At the point of transcription, interviews were transcribed verbatim with all identifying details removed to ensure anonymity. We followed the analytical framework defined by Braun and Clarke (2006) to undertake a thematic analysis of data. We read all transcripts multiple times, coded two transcripts independently and then agreed on a coding framework, while maintaining openness and continuous dialogue throughout the analysis process. The software package NVivo 12 was used to undertake the analysis. All data were stored in an encrypted digital store and interview recordings were destroyed once their transcription was complete.

## Findings

Participants were aged 30 to 38 at the time of the interview; the average age was 33.9 years. 13 participants described their gender as female, and one described his gender as male. All participants were of White or White British ethnicity, and their household income was in all cases above the threshold of £30,000 per year, with an average annual household income of £54,000. 12 participants resided in Scotland, and two participants resided in England.

All 14 parents attended parent groups before and during the lockdown and all but one parent was actively involved at the time of the interview. Seven parents were enrolled in online paid sessions including baby sensory, music and dance, baby massage, postnatal yoga, breastfeeding support, songs, rhymes and stories. 13 parents were part of peer support networks based on Facebook or WhatsApp chats, and 12 were active members of a range of unpaid online groups and networks. Two parents accessed support for their mental health, both of whom were already receiving or requesting support before the lockdown, and eight parents said they were in contact with their health visitor, midwife and/or nurse at the time of the study.

Four overarching themes guided the interpretation of findings: (1) The virtual parenting community as a ‘panacea’; (2) Making do with online classes; (3) The need for the embodiment of connection; (4) Increased awareness of self-presentation and impression management.

### The virtual parenting community as a ‘panacea’ 

The lockdown period was described as ‘intense’: parents felt isolated and questioned whether their parenting was right or good enough and, without the support and interaction with other parents or family members, found this hard to gauge. ‘Rose’ expressed her frustration with the lockdown situation clearly.

‘I’m trying not to go mad.’ [Rose, mother of a 6-month-old]^1^

Participants anticipated that their parental leave would be spent bonding with their baby and other parents; they highlighted the importance of the social aspect of parental leave and their pre-COVID-19 intention to spend time with family and meet other parents. ‘Jenny’ expressed a sense of betrayal that many of these expectations were denied due to the physical distancing restrictions and felt connected to other mothers sharing her same experience.

‘I sound selfish when I say this, but I feel like this whole virus has kind of taken away my maternity leave, and I totally understand and feel sorry for everybody this is happening to in the world.’ [Jenny, mother of a 7-month-old]

The experiences of parenting during lockdown highlighted the essential role of structured group support. Some of the groups had connected WhatsApp group chats which allowed ‘mummy friends’ to sustain pre-existing connections and establish new relationships. Some participants had met other parents and their babies in groups before lockdown, which was found to be a relief for parents as they had other parents to share experiences with despite not being able to connect in person.

‘We’ve already met [other parents] in person. So, you feel like you’ve already established that relationship.’ [May, mother of a 3-month-old]

Parents found the support and entertainment of WhatsApp chats with parents with similar-aged babies a light relief as they shared memes, milestones and funny stories. These chats were described as an extension of the structured online groups, and this package was treated as a survival mechanism or even a panacea for parents. Mothers and a father (in this sample) were gratified by the opportunity of getting ‘immediate answers’ from chats affiliated with parent groups. Parents navigated their journey together, shared their personal experiences and offered advice to others to cope with everyday and extraordinary challenges. For ‘Lily’, parent group chats were already a staple feature of her parental leave before the pandemic and gave her both joy and connection.

‘We were pinging messages all the time. We were having hilarious chats about Pat Sharp and Fun House and 80s TV shows, just being there together. My baby had a circumstance where he didn’t poop for 8 days. So, we were on “poo watch”. […] It was just safe, non-judgemental.’ [Lily, mother of a 1-year-old]

‘Jenny’ and other parents described the unique opportunities arising from the chance to speak with peers about their struggles. Group chats, therefore, provided an incredible source of emotional and practical support.

While attending online groups, ‘Jenny’ describes below how she felt able to hold space for other mums who needed extra support. ‘Paul’ confirms that a fathers’ group gave him a similar sense of containment.

‘One of the girls today was talking about how her week was going. She got really upset and we were kind of like - that’s why we’re here, to speak with you, and she’s like, I know that everybody’s got their problems, but it’s why we’re there, you know, to share them. […] If you’re feeling down make sure you open up to another mum, ‘cause it’s totally amazing the response and the support you get from other mums.’ [Jenny, mother of a 7-month-old]

‘I think for guys it’s quite nice to have a group of guys that are also going through their fatherhood. So yeah, I suppose if you really had an issue, I think in these groups you could probably find someone to speak to about what you needed to find out.’ [Paul, father of a 1-month-old]

Online parent communities were deemed more valuable if they involved multifaceted, flexible opportunities for participation, and living close by to other parents was a way to extend relationships. The most structured networks included baby groups, as well as parent forums on Facebook and WhatsApp and membership was influenced by social circles, location or the babies’ age. Sharing experiences and struggles were encouraged, and according to ‘Lily’, her parent group even had its own economy.

‘We have a WhatsApp group and chat to each other and like we don’t even talk about money anymore. We have our own currency called “swap currency”. So, like yesterday [a ‘mum’ friend] got me bubbles in her online order so we can have them for [baby]’s party, and I didn’t give her the £3 and I won’t give her the £3, but I might be downtown and she might need some milk and I’ll buy that.’ [Lily, mother of a 1-year-old]

Although online parent communities were a much-needed social lifeline for most parents in our study, parent and baby classes did not always have the same success when translated to an online medium.

### Making do with online classes

Alongside parent chats, online groups helped build a routine and created special occasions to look forward to during the pandemic. Group location was no longer an issue, as some parents could engage in sessions that were usually too far away to attend. Some sessions were pre-recorded, and parents could access them at their leisure while following their babies’ schedules.

Some parents praised the efforts made by group facilitators to entertain the babies, adapting synchronous activities to the families’ routines and keeping the usual in-person group activities such as ‘check-in’ conversations at the start of the sessions. ‘Claire’ and ‘Ann’ described the sense of structure that the virtual groups provided to their lives in lockdown, as they provided a much-needed regular chance to socialise with peers.

‘I think just having that group of mums that are exactly at the same stage is invaluable, and I think it’s giving me a routine ‘cause you know you go along the same time every week. You have your little baby class, and everyone stay for, like a cup of coffee.’ [Claire, mother of an 8-month-old]

‘When you’re on your own for the whole time, you kinda just want a little bit of escapism’. [Ann, mother of a 7-month-old]

However, certain groups could not be replicated online due to the inaccessibility of props and facilities, such as soft play or swimming. Technology glitches meant that some sessions were unavailable or that sound and visual quality were compromised. Overall, reviews of online classes were mixed and parents’ motivation to continue fluctuated. Some babies got easily distracted from screen-only experiences that could not reproduce the sensory experience of the class with movement and interaction, and parents questioned whether their children were getting anything from participating.

Due to their multisensory nature, some classes, such as baby massage, yoga, and songs and rhymes, remained effective online. Babies were also attracted by seeing faces and compelling animations such as lights and bright colours and having the class ‘cast’ onto television was beneficial. However, using the laptop or tablet could be hazardous with babies reportedly pressing buttons and pulling wires. ‘Maggie’ expresses her contentment with taking part in a remote yoga class that benefitted both her and her baby, to the point it changed the course of a day that would have otherwise been difficult.

‘I feel so much calmer after [yoga] and I feel my body feel so much better after it as well. Like, I can tell the difference in a week that I haven’t done it. So, I’m definitely fitter and happier from doing it which impacts on her, so Monday doesn’t seem to be like a grumpy day.’ [Maggie, mother of a 6-month-old]

Participants reported that some group facilitators did not feel comfortable with online facilitation and had suspended their groups. Other participants engaged in creative efforts to offer online groups and suggested resources to enable parents to replicate activities at home, such as using cooking utensils as substitute musical instruments. Although these endeavours were appreciated, ‘Becca’ was aware that reproducing the leaders’ routines or building props with recycled materials was often inadequate and led to increased labour for her as a mother.

‘It’s a bit different because you’re more of an observer when you’re in a [in-person] class. You know, whereas when you’re in the house you’re the one doing all the dancing about and the shaking and the singing trying to get her to engage, whereas you can watch her just watching everything else when you’re in the class.’ [Becca, mother of a 6-month-old]

Finally, for some parents, there was a sense of injustice that the cost of online group activities was not reduced, and after trying online classes for a block of 6 weeks or so, some parents decided not to continue. Further disappointment with the online classes emerged from the inevitable lack of shared physical intimacy amongst other parents.

### The need for the embodiment of connection

While the online groups could be accessible to larger populations as there were no time or space restrictions, parents missed the intimacy of smaller groups, particularly the richness of face-to-face interaction. During lockdown, digital technologies became essential for people seeking to maintain relationships and participate in parent and baby groups. Yet, parents expressed concerns about the dearth of spontaneous conversations, as only a glimpse of reality was present, and conversations were made unnatural by the need for conservation turn-taking, being sometimes literally ‘muted’ by the group facilitator, as ‘May’ accounts. ‘Una’ was candid about her experience, as interacting with facilitators and other mothers seemed alienating and mechanical in online spaces.

‘You’re missing the in-person cues…and it is like talking to very good robots, it’s probably the best way I can put it.’ [Una, mother of a 5-month-old]

Parents often could not talk informally with other parents and their babies before, during and after sessions and had to ‘raise their voice’ or emoji hand, if they wanted to be heard, as ‘Una’ noted above; interactions became mono or bidirectional rather than multidirectional. Informal spaces to share spontaneous and private conversations lacked, and these omissions highlighted what was important to parents: the need for the embodiment of connection. ‘Lily’ was clear about her need to feel grounded by connecting physically with other parents and babies sharing the same space or in ‘Becca’s’ experience noted earlier; observing her baby observing the world.

‘Physical contact is so important, not just like physically touching people, like being in the same space is so important when you’re a new mum.’ [Lily, mother of a 1-year-old]

‘Maggie’, a mother of a 6-month-old, reminisced about the luxury of in-person groups and the opportunities for bonding with other parents after sessions.

‘…Maybe afterwards you go off and having a bit of a walk around town with one of the other mums. I’m really missing that. So, then you could have like more kind of private chats about, you know. About “Are you having sex again?” Yeah, those things that you maybe don’t want to talk about with many of the people you see.’ [Maggie, mother of a 6-month-old]

Some group facilitators made a concerted effort to help parents connect and talk beyond the class setting, recognising their need for informal support. This happened by creating new class WhatsApp groups or reserving extra time for open conversation without the facilitator. As ‘Jenny’ narrated, some mums were able to share intense emotional moments on camera.

‘We had class today and after the class, even during the class they asked, “how’s your week going”, and we were all crying, oh we were just so emotional...’ [Jenny, mother of a 7-month-old]

However, the opportunity to open up freely involved a risk of ‘oversharing’, with the potential of the class overrunning as ‘Lily’ highlights.

‘The chat was really good as well at the beginning, but some people I felt hogged it and went and talked for too long and you’re a bit like, “Right, OK stop”. You need to like give everyone space, especially ‘cause we’re paying for this class. I’m not paying for therapy.’ [Lily, mother of a 1-year-old]

### Increased awareness of self-presentation and impression management

Parents engaged in various self-presentation strategies. Parents could mute their microphones whenever their babies cried, turn their camera off or leave the screen space if they needed to breastfeed, increasing their sense of comfort and control of the virtual space. Online social engagement carried idiosyncratic meanings regarding how ‘display work’ was done in an altered social environment. Some parents were conscious that they and others might feel judged for having their living spaces on public display; in this sense, they perhaps hinted at the existence of a ‘protective cocoon’ to protect themselves from what was perceived as a judgemental gaze. Nonetheless, caring about self-presentation, namely, parent’s own appearance was framed as a means of establishing a sense of normality, as ‘Claire’ explains.

‘I think the good thing is that they give you a routine, so whenever I’m doing the paid [groups], I make sure that I’m dressed up, I put my make-up on, get the baby all dressed...So that’s important because you feel that you’re, just…it’s normal.’ [Claire, mother of an 8-month-old]

In her voluntary role as a group facilitator, ‘Una’ was straightforward on the inevitability of judgement and suggested agreeing on a ‘suspension of disbelief’ to help parents feel safe and focus on mutual support.

‘The only thing we could do is sort of say to people, “I don’t care what your house looks like, I don’t care how noisy your children are”. People might say this is a safe and non-judgemental space. And you’re like, no it’s not, everybody judges, let’s not pretend that we don’t, let’s just say we don’t care. I don’t have capacity in my head to judge your house. I’m too busy judging my own.’ [Una, mother of a 5-month-old]

It is clear from our findings that online networks and groups were useful and offered a sense of routine that supplemented their parenting experience. Amongst other benefits, there was also a sense that connection and intimacy was lost in online settings, and that cues of distress could be hidden or minimised. ‘May’, a new parent and social worker, was concerned that the absence of a shared physical space for new parents meant there was less opportunity for parents to reach out for help and for other parents and group facilitators to know when to offer support.

## Discussion

This study aimed to learn about new parents’ experiences of online groups during the first COVID-19 lockdown. Consistent with literature about the pandemic (Molgora and Accordini, 2020), participants in our study experienced isolation in lockdown, although to some extent, they were able to connect at a deeper level with their closest family members in the household. Groups provided parents with entertainment, support and routine and were effective when activities could be replicated at home, such as exercise or multi-sensorial stimulation. There was an overriding preference for the opportunity to foster and create meaningful relationships with other parents at a similar stage of parenting in group settings. However, turn-taking and the lack of physical space, tended to impair the sense of intimacy and connection in online groups.

In the short term, at the start of the lockdown period, online groups supported parenting experiences. However, there was a sense that connection and intimacy was lost in online settings, and that cues of distress could be hidden or minimised. There was also a demand for the return to physical classes. For example, lobbying led to a U-turn in Scotland and organised activities re-commenced (Scottish Government, 2020).

This study supports existing pre-pandemic literature about the benefits of groups for new parents (Madge and O’ Connor, 2006). Parents valued group activities for entertainment and stimulation, to share everyday stories, reduce social isolation and develop connections. The wider virtual parenting community was also highly regarded; chats linked to groups, on Whatsapp and Messenger were an essential source of emotional and practical support which created a strong sense of community. Many parents used informal networks or even time within group sessions to seek support for parenting experiences, including breastfeeding and mental health support.

This study suggests that parents may have greater control over self-presentational behaviour in a virtual setting and manage their online interactions more strategically in comparison to in-person settings. In regard to self-presentation, the most significant tension experienced by participants was not unique to the online medium: mediating between the pressures to present an ‘enhanced’ or ‘desired’ self (Goffman, 1959). Comparisons to other parents and babies can be useful to mark progress and identify distinctiveness, but also overwhelming and may elicit feelings of inadequacy. The opportunity to control what is on display can be regarded as a positive self-presentation strategy for users, yet a potential barrier for providers to identify signals for help.

### Recommendations for policy and practice

However, the reception of online support can be evaluated by adult consumers, it was not possible in this study to examine babies’ experiences and perceptions. There is consensus from experts that play and interaction with humans are essential for children’s development and should not be replaced by technology-mediated play (Kucirkova and Sakr, 2018). However, the longer-term benefits and disadvantages of online mediated support for parents and, infants remain to be seen. Following Liu, Erdei and Mittal (2021), healthcare providers may consider inquiring about the emergent emotional experiences that have arisen from the pandemic, including worries or feelings of grief due to the various losses that occurred.

Hybrid methods of group work are recommended as a way forward in the delivery of groups for new parents. It is recommended that parent classes continue face-to-face, whenever possible, but there is the chance to access recorded or pre-recorded resources if parents are unable to participate. The virtual parenting community was found to be a significant way to supplement groups, as they provide opportunities for connection, immediate support and advice on everyday issues.

While no data exist on the retention and/or attrition rates for online groups during the pandemic, there are a number of suggestions for group faciltitators. Online groups can increase accessibility, by attracting people from beyond the immediate area, but service providers should be aware that parents may not know about the existence of such groups and as such, a current resource list with links to groups, may be useful for parents, perhaps distributed during routine Health Visitor appointments.

If in-person groups are not feasible due to public health restrictions, the possibility of free online groups and grants for facilitators and businesses, to promote parent and babies’ health, wellbeing and connectedness should be investigated. As a participant suggested, private online classes could support formal healthcare provision, for example, sharing reminders of routine developmental checks, as well as providing ways to access formal support. Support for online group facilitation should also be provided to enable businesses and practitioners to effectively provide services. Specialist training and guidance could be helpful to support facilitators to explicitly address the need to create welcoming environments where parents can show their vulnerability without the fear of judgement. The need for intimacy in online groups could be addressed by making sure that parents can speak privately with the facilitator or meet in smaller ‘break-out’ rooms with their peers.

### Study considerations and future research

The small sample and demographic characteristics of participants do not allow for the generalisation of findings to the general population. In the analysis process, we questioned the lack of diversity that emerged from this study’s sampling strategy. Participants’ recruitment happened within parent social networks that were geographically close and known to the researchers, which likely affected the demographic of the sample. The sample was based on who responded to the advert; although it was not in the researchers’ intent to exclude non-White British participants or parents from low-income families, the recruitment strategy was not able to reach these populations. Further research should explore whether there are accessibility issues in parent groups and if these have an influence on the participation of fathers, minoritised parents or low-income families in support networks or if any specific barriers determined their exclusion from this study. It would also be worth investigating whether fathers and parents from minoritised or low-income families tend to access support networks unknown to the researchers.

The online medium likely impacted not only parent and service providers’ relationships but also the research setting itself: not being able to conduct interviews face-to-face increased participants’ accessibility to the study, as they participate without leaving their homes and their childcare duties, but this might have had an impact on their sense of intimacy and therefore the depth of the conversations. Follow-up, reflective interviews with the participants might explore their perceptions of ease during the study, with potential benefits for further remote research.

This exploratory study contributes to an underrepresented area of research by drawing a portrait of how new parents in the United Kingdom experienced technology in their early parenting journey. Future research could capture multiple perspectives, from parents and group facilitators and to evaluate the benefits for parents and babies.

## Conclusion

This study illustrates how a sample of new parents experienced online sources of support during the first COVID-19 lockdown, including virtual parent chats and remote classes or support groups. It is innovative as it shows the hidden stories of new parents interacting and co-creating virtual communities in lockdown, shifting their sense of connection. While online technologies are ubiquitous, online groups for parents are nascent, and these technologies require careful analysis and evaluation from users and organisers.

## Note

1. All names have been substituted with pseudonyms to protect participants’ privacy.
